# A Computationally Designed Prefusion Stabilized Human Metapneumovirus Fusion Protein Vaccine Antigen Elicited a Potent Neutralization Response

**DOI:** 10.3390/vaccines13050523

**Published:** 2025-05-15

**Authors:** Michael Kishko, Antonia Stuebler, Sukanya Sasmal, Yvonne Chan, Dean Huang, Christopher Reyes, Jasmine Lin, Owen Price, Ana Kume, Katie Zong, Christine Bricault, Judith Alamares-Sapuay, Linong Zhang

**Affiliations:** Sanofi, 200 West Street, Waltham, MA 02451, USA; michael.kishko@sanofi.com (M.K.); antonia.stuebler@sanofi.com (A.S.); sukanyasasmal@gmail.com (S.S.); yvehchan@gmail.com (Y.C.); dean.huang@sanofi.com (D.H.); christopher.reyes-giler@sanofi.com (C.R.); jasmine.lin@sanofi.com (J.L.); owen_price@hms.harvard.edu (O.P.); ana.kume@sanofi.com (A.K.); katie.zong@sanofi.com (K.Z.); christine.bricault@sanofi.com (C.B.); judith.alamares-sapuay@sanofi.com (J.A.-S.)

**Keywords:** computational design, hMPV, subunit vaccine, prefusion stabilization

## Abstract

**Background/Objectives:** Human metapneumovirus (hMPV) is a leading cause of respiratory infections in the elderly, with high morbidity and mortality and with no vaccines or specific therapies available. The primary protective antigen of hMPV is the fusion protein, and its prefusion conformation (pre-F) is considered the most promising target for vaccine development. **Methods:** Utilizing computational design strategies focused on intraprotomer interface stabilization, we designed hMPV pre-F recombinant subunit vaccine candidates based on the most prevalent A2 subtype and characterized them in vitro and in vivo, benchmarking to the prototypical hMPV pre-F stabilized by an introduction of a proline at site 185. **Results:** The top candidate (N46V_T160F) yielded 14.4 mg/L with a melting temperature of 79.3 °C as compared to 5.7 mg/L and 70.4 °C for the benchmark. By employing monoclonal antibody binding to all six antigenic sites of hMPV pre-F, we confirmed this construct retained all pre-F specific antigenic sites and that the key sites Ø and V were stable at 4 °C for up to 6 months. When immunogenicity of N46V_T160F was evaluated in mice, it induced higher binding and neutralizing antibody titers than the benchmark, which stemmed in part from increased levels of site Ø and site II targeting Abs. Further, this A2 based construct induced cross-neutralizing Abs against all four hMPV subtypes. Lastly, our construct exhibited similar immunogenicity as the recently published next-generation hMPV pre-F constructs, DS-CavEs2 and v3B_Δ12_D454C-V458C. **Conclusions:** N46V_T160F is a promising hMPV vaccine candidate paving the way for further development and optimization.

## 1. Introduction

Human metapneumovirus (hMPV) is a respiratory pathogen associated with high morbidity and mortality in older adults with no vaccines or specific therapies available [1,2,3,4,5]. While all individuals are exposed to hMPV by the age of five [6,7], with a significant disease burden in this population [8], repeated exposures do not protect adults against re-infection [3,9]. HMPV is a member of the Pneumoviridae family of negative-strand RNA viruses which encode three surface expressed membrane proteins. Of these, the fusion (F) protein mediates entry [10], is the major target of the humoral immune response [11,12], and is the main focus of vaccine development efforts [13,14,15].

The Pneumoviridae family also contains the closely related respiratory syncytial virus (RSV). The F protein of RSV likewise mediates entry and is a major target of the humoral immune response and the main focus of vaccine and therapeutics development efforts [10,16,17]. Recent RSV vaccine development is guided by high-resolution 3D structures showing that, similar to hMPV, its F protein exists in two distinct conformations, a metastable prefusion (pre-F) [18] and stable post-fusion (post-F) [19]. Of these, pre-F is more effective in inducing neutralizing antibodies (nAbs) [20,21]. The three recently approved RSV vaccines for older adults all utilize pre-F antigens [22,23,24], while the highly potent monoclonal antibody Beyfortus (Nirsevimab) recently approved for prevention of RSV lower respiratory tract disease in infants during their first RSV season targets a pre-F specific epitope [16].

Similarly, it was shown that hMPV F exists in pre-F and post-F states [25,26]. HMPV F is a class I viral fusion protein that is initially translated as the F0 single chain precursor that is proteolytically cleaved by extracellular, trypsin-like, serine proteases (or by trypsin in tissue culture) into the disulfide linked F1 and F2 subunits. Three F1–F2 protomers then associate to form the metastable pre-F trimer. During fusion, conformational changes allow a hydrophobic sequence termed the fusion peptide, located at the N-terminal of the F1, to insert into the host-cell membrane, followed by transition to the highly stable, 6-helix bundle post-F state. Immunization of mice with hMPV pre-F induces significantly higher nAb titers than post-F [11,27]. Therefore, similar to RSV, pre-F stabilization is proposed as a means to produce vaccine antigens capable of inducing high titers of nAbs against hMPV [11,28].

There are two genetic lineages of hMPV, A and B, that are antigenically distinct and that are further divided into the four primary subtypes (also referred to as subgroups or sub-lineages) A1, A2, B1, and B2 [29]. In recent years, detection of A2 predominated, followed by B1 and B2 at lower prevalence levels, while subtype A1 has not been detected in recent years [29,30,31]. HMPV phylogeny is commonly based on the attachment protein (G) sequences. The amino acid identity of G between the lineages is only 30–37% [32], and G does not elicit neutralizing antibodies [12,33]. While the maximum diversity between the hMPV F proteins at the amino acid level is only ~6% [34], the A and B lineages are antigenically distinct via the classic virological definition of >16-fold difference between homologous and heterologous neutralizing antibody (Ab) titers [32].

Towards designing a recombinant subunit hMPV F vaccine candidate, we utilized computational design in combination with available structural data to explore three strategies to stabilize hMPV F in the pre-F state, including hydrophobic cavity filling and intraprotomer and interprotomer interface stabilizations. We introduced these mutations into the F of the CAN97-83 prototypical strain from hMPV subtype A2, which our analysis showed to be the subtype most prevalent in the last decade. The most promising candidate was the intraprotomer design N46V_T160F. This candidate maintained all antigenic sites and exhibited a 8.9 °C higher melting temperature than a pre-F benchmark generated by introducing proline 185 into our base sequence, following the prototypical hMPV pre-F stabilization strategy from the McLellan laboratory [25]. In mice, N46V_T160F was more immunogenic than the benchmark, with enhanced neutralization stemming at least in part from increased levels of site Ø and site II targeting Abs. Sera from N46V_T160F immunized mice potently neutralized prototype strains from all four hMPV subtypes, and demonstrated similar induction of binding and neutralizing Ab titers as the recent, next-generation, structure based, hMPV pre-F designs v3B_Δ12_D454C-V458C [27] and the DS-CavEs2 [11].

## 2. Materials and Methods

### 2.1. Computational Design

The ectodomain sequence from the A2 subtype isolate CAN97-83 (GenBank accession no. AY145296) was selected as the base sequence for our designs based on circulating prevalence. Briefly, we performed a GenBank search for hMPV F genes and analyzed the amino acid sequences, focusing on prevalence over time and sequence similarity (Figure S1). Subtype A2 was predominant in most of the recent years analyzed.

To stabilize the pre-F conformation, we selected mutations based on the ΔΔG of stability computed using the Rosetta modeling software version 2021.07 [35]. The mutations were generated based on three biophysical strategies: cavity filling and interprotomer and intraprotomer interface stabilization. For cavity filling mutations, we mutated selected residues into large hydrophobic residues such that the core of the protein was stabilized, and in turn, the pre-F conformation was more stable. For interfacial stabilizations, we identified residues which interact only in the pre-F conformation and aimed to increase the interaction strength by either introducing stronger hydrophobic or electrostatic interactions.

After generating the designs, we used Rosetta ΔΔG calculations to rank the designs. We calculated the free energy difference between the pre-F and the post-F conformations of the proteins for the mutants and the wild type. The pre-F and the post-F conformations were modeled based on the PDBs 5WB0 [25] and 5L1X [26], respectively. The Rosetta protocol for the ΔΔG of stability consisted of a Rosetta relaxation step and a Cartesian ΔΔG [36] calculation step. The atomic coordinates present in the PDB structure were first relaxed in the Cartesian space using the scoring function ref2015_cart to remove any unfavorable contacts. We minimized the initial structure for twenty iterations and then selected the structure with the lowest energy for the next ΔΔG calculation step. The ΔΔG values were calculated by averaging over three iterations of Cartesian ΔΔG calculations. We used the beta_cart scoring function and 9.0 and 1 for the fa_max_dis and bbnbr parameters, respectively. In the last step, we selected mutations based on the ΔΔG metric. Additionally, since we had a limited experimental testing capability, we diversified the residue positions being tested by selecting only the top design for each residue position.

Other modifications to the base sequence included modification of the canonical cleavage site and C-terminal modification for formation of stable soluble trimers. The “RQSR” cleavage site between the F1 and F2 subunits was replaced with a trypsin-independent furin protease motif “RRRR” [25]. To convert the membrane bound, surface expressed, hMPV F to a soluble, purifiable version, the base sequence was truncated at the C-terminal end of the ectodomain, removing the transmembrane domain and the cytoplasmic tail. These were replaced with a flexible GS linker (GGGS), a T4 foldon trimerization domain [37], a HRV-3C protease site, a flexible “G” linker, and the purification tags 8XHis and StrepII.

In addition, three benchmarks were included. The first was the prototypical pre-F A185P construct from the McLellan laboratory, which was based on an A1 subtype strain and stabilized in the pre-F conformation via the introduction of a proline “helix breaker” residue [25]. As we produced this construct in its native subtype A1 strain background, to permit the most direct comparison with our designs we also introduced a proline into the corresponding location of the A2 subtype CAN97-83 strain F, generating the D185P construct. Lastly, the subtype-A1-based post-F construct from the McLellan laboratory was engineered [26].

### 2.2. Protein Expression and Purification

All hMPV designs, containing a trimerization domain and a His- and a StrepII-tag, were constructed into plasmids and verified by DNA sequencing. Plasmids encoding the target protein and furin, unless otherwise stated, were used to co-transfect HEK293 cells (Sino Biological, Beijing, China) at a 4:1 ratio in a total culture volume of 1 L. The transfected cells were cultured at 37 °C with 8% CO_2_ for 6 days. After this period, the culture supernatant was collected for protein purification. The hMPV constructs were immobilized using metal affinity chromatography (IMAC). The His-tagged proteins were eluted in a buffer containing 50 mM Tris and 150 mM NaCl at pH 7.4. If necessary, a second purification step involving gel filtration was performed using a Superdex 200 PG column (Cytivia, Marlborough, MA, USA).

### 2.3. Size Exclusion Chromatography (SEC) and SEC Coupled with Multi-Angle Light Scattering (SEC-MALS)

SEC was used to assess trimer percentage and performed using an Agilent-1100 with a TSK G3000SWXL column and a mobile phase consisting of 0.2 M NaH_2_PO_4_, 0.1 M Arginine and 1% IPA at pH 6.5. Baseline correction was performed and resulting peaks were integrated to report approximate trimer percentage. SEC-MALS was performed using a Waters Arc Bio system containing a UV detector coupled to a μDAWN light-scattering detector and an Optilab UT-rEX refractive index detector (Wyatt Technology, Santa Barbara, CA, USA). A total of 20–40 µg of purified protein was passed over a 1.7 µm, 200 Å BEH SEC column (Waters Corporation, Milford, MA, USA) at a constant flow rate of 0.3 mL/min with 50 mM Tris and 150 mM NaCl at pH 7.4. Empower software version 3.8.0 (Waters Corporation) was used to control the chromatography system, and Astra (Wyatt Technology) was used for data collection and analysis. Monomeric bovine serum albumin (BSA) was used to normalize the light-scattering detectors. Data were processed to determine the weight–average molar mass and polydispersity of the protein sample using the Debye model.

### 2.4. Nano Differential Scanning Fluorimetry

Purified hMPV constructs were diluted in formulation buffer (50 mM Tris, 150 mM NaCl, pH 7.5) to 0.5 mg/mL and loaded into High Sensitivity Capillary Chips (NanoTemper Technologies, Munich, Germany). All measurements were performed using a Prometheus (NanoTemper Technologies). Excitation power was set to 30%, and the samples were heated from 20 to 95 °C at a rate of 1.5 °C/min. Data were recorded with the PR.ThermControl software version 2.1.6 (NanoTemper Technologies) and analyzed using PR.Stability Analysis v1.01 (NanoTemper Technologies).

### 2.5. Linear and Conformational Antibody Binding Analysis by Biolayer Interferometry

Conformation of the various hMPV constructs was determined using an Octet HTX system (Sartorius, Göttingen, Germany). All samples and antibodies were diluted in kinetic buffer (Sartorius) to a 1X dilution with phosphate buffered saline (PBS) to 5 µg/mL and 1 µg/mL, respectively. Antibodies were loaded onto Protein A biosensors, and the binding of all antigens was tested under the following conditions: initial baseline in kinetic buffer alone (120 s), loading of the various antibodies (180 s), second baseline in kinetic buffer alone (120 s), followed by dipping into wells with the antigen only for the association of the antigen (180 s), and dissociation of the antigen in wells with kinetic buffer alone (120 s). Binding results were analyzed using ForteBio Data Analysis 12.0 software (Sartorius).

### 2.6. Viruses and Cells

Vero (African green monkey kidney epithelial cells, ATCC CCL-81, Manassas, VA, USA) cells were obtained from the American Type Culture Collection and propagated in Dulbecco’s Modified Eagle Medium (DMEM) supplemented with 10% fetal bovine serum (FBS), L-glutamine, and antibiotics. Recombinant hMPV CAN97-83 (subtype A2) expressing the GFP reporter was obtained from ViraTree (Durham, NC, USA, Product # M121). Wild type hMPV strains NL/1/00 (subtype A1), NL/17/00 (subtype A2), NL/1/99 (subtype B1), and NL/1/94 (subtype B2) were obtained from the laboratory of Bernadette van den Hoogen at the Erasmus Medical Center [32]. All viruses were propagated in Vero CCL-81 cells using media supplemented with 5 µg/mL trypsin and titrated using a plaque assay, essentially as described for RSV [38], except that the overlay was supplemented with 5 µg/mL trypsin, growth under the overlay was performed at 32 °C, and the primary and secondary detection Abs were respectively Anti-Metapneumovirus fusion protein antibody (ab94800) and Rabbit Anti-Mouse IgG H&L (HRP) (ab6728) (Abcam, Waltham, MA, USA).

### 2.7. Mouse Immunogenicity Studies

The studies were carried out in strict accordance with the recommendations in the Guide for the Care and Use of Laboratory Animals. The protocols were approved by the Sanofi Institutional Animal Care and Use Committee (Animal Use Protocol (AUP) Number 20/0424). Mice for the first study were obtained from Charles River Laboratories (Wilmington, MA, USA) and for the second study from Jackson Labs (Bar Harbor, ME, USA), co-housed at 4 mice per cage, and monitored daily for any signs of pain or distress as specified in the AUP.

In the first study, hMPV F proteins were mixed with Alhydrogel adjuvant (Brenntag Northeast, Inc., Reading PA, USA) at a 1:1 antigen to adjuvant volume ratio. Groups of eight female, 6- to 8-week-old, BALB/c mice were intramuscularly immunized with 50 μL per hind leg of the antigen and adjuvant mixture containing a total of 0.5 µg of protein per dose. The mice received 2 doses, 21 days apart. Blood was collected prior to, and 35 days after, the first dose.

As the hMPV post-F benchmark was reported to be a mixture of both pre-F and post-F [26], it was heat treated at 70 °C for 10 min to fully convert it to the post-F state [25] prior to inoculation into mice.

The second study was conducted identically to the first, except that the Alhydrogel adjuvant was obtained from InvivoGen (San Diego, CA, USA), and the mice were bled one day prior to, rather than just before, the first immunization.

### 2.8. Enzyme Linked Immunosorbent Assay (ELISA)

ELISAs were performed essentially as described in [39], except that plates were coated with the hMPV subtype A1 based pre-F A185P for the first mouse study (as this was the only fully characterized pre-F antigen then available), and with the A2 based pre-F N46V_T160F for the second mouse study, where all pre-F constructs were A2 based.

### 2.9. Competitive ELISA

Serum-site-specific Immunoglobulin G (IgG) titers were determined through competition with monoclonal antibodies (mAbs) using ELISA. The mAbs were SAN32-2 to antigenic site Ø [40], DS7 to site I [41], and 338 to site II [42], and all were biotin labeled. Briefly, microtiter plates were coated with 100 ng per well of hMPV pre-F N46V_T160F in PBS and incubated overnight at 4 °C. Coated plates were washed with PBS + 0.5% Tween 20 (PBST) and blocked with PBST + 1% BSA for 1 h at 37 °C. The blocking solution was discarded, and sera were two-fold, 10-point, serially diluted in fresh blocking solution in the coated plates. After a 90-min incubation at 37 °C, the plates were washed with PBST and one of the biotin-labeled, site-specific, mAbs was added to the plate at dilutions previously determined to result in an optical density (OD) of ~1.3 in the absence of competing sera. The plates were incubated at 37 °C for 30 min and washed with PBST and Horseradish Peroxidase conjugated α-Biotin Ab (PA1-30595, ThermoFisher Scientific, Waltham, MA, USA) was then added for 1 h at 37 °C. The plates were washed with PBST and developed with 3,3,5,5-tetramethylbenzidine (TMB) substrate solution (ThermoFisher Scientific, Waltham, MA, USA) for 6 min. The colorimetric reaction was stopped with Thermo Scientific™ Pierce™ Stop Solution for TMB (ThermoFisher Scientific, Waltham, MA, USA) and ODs were measured at 450 nm.

To calculate the 50% inhibitory concentrations (IC_50_), the average OD from 8 wells that did not receive test sera nor competing mAbs (background) was subtracted from all other wells on the plate. The background subtracted OD Max for each plate was calculated as the average from 8 wells that received competing mAbs but no sera. The percent inhibition in each serum test well was then calculated using the formula “100 − (OD in well * 100)/OD Max”. Any negative numbers returned by the formula were set to 0, and the IC_50_ of log transformed values were interpolated via 4-parameter logistic regression in GraphPad Prism software version 10.2.3 (Dotmatics, Boston, MA, USA).

### 2.10. Microneutralization Assays

Vero-81 cells were seeded at 64,000 cells/well in 96-well plates suitable for fluorescence reading one day prior to infection. Serum samples were heat inactivated and 4-fold serially diluted from 1:20 to 1:81,920 in FluoroBrite DMEM (ThermoFisher Scientific, Waltham, MA, USA) supplemented with L-glutamine and antibiotics (assay media). Diluted sera were combined 1:1 with the hMPV-GFP reporter expressing virus from ViraTree, diluted in assay media, and incubated for one hour at room temperature. The serum-virus mixtures were then added to the cell plates and incubated for 24 h at 37 °C. The plates were read on a high content imager and the fluorescent events were quantified. Serum 50% neutralizing titers were calculated using 4-parameter logistics regression in the SoftMax Pro software version 6.5.1 GxP (Molecular Devices, San Jose, CA, USA).

### 2.11. Plaque Reduction Neutralization Test

Neutralization of wild type viruses was assessed via a 60% Plaque Reduction Neutralization Test (PRNT_60_). Briefly, Vero cells were seeded at 160,000 cells/well in 500 μL in 24-well plates one day prior to infection. On the day of infection, serum samples were heat-inactivated and initially diluted 1:20, and further four-fold, 5-point, serially diluted in virus growth medium (VGM) consisting of OptiPRO™ SFM (ThermoFisher Scientific, Waltham, MA, USA) supplemented with 1% Penicillin–Streptomycin and 2% FBS.

HMPV stocks were diluted to 2000 Plaque Forming Units (PFU) per mL in VGM, added at 1:1 ratio to the diluted sera, and the mixture was incubated for 1.5 hours at 32 °C and 5% CO_2_ to allow neutralization to occur. The medium was then removed from the cell plates and the Vero cell monolayers were infected with 100 μL/well of serum/virus mixture (targeting ~100 PFU/well) for 1.5 h at 32 °C and 5% CO_2_, with gentle rocking every 15 min. At the end of this incubation, 1 mL of 0.75% methylcellulose overlay supplemented with 5 µg/mL trypsin was added to each well. Plates were subsequently incubated at 32 °C and 5% CO_2_ for 6 days to allow plaques to develop, then fixed and immunostained, as described for the viral titrations. Plaques were counted using a dissecting microscope and 60% plaque reduction neutralizing titers were calculated in Excel (Microsoft, Redmond, WA, USA). Four replicates of an in-house generated adult human sera pool were tested in each assay as an intra- and inter-assay control and neutralization benchmark.

### 2.12. Statistical Analysis

Day 35 microneutralization titers from the mouse immunogenicity study were log_10_ transformed and analyzed using One-Way Analysis of Variance (ANOVA) with groups as fixed factors and a Tukey adjustment. Mouse immunogenicity ELISA titers were log_10_ transformed and analyzed using Two-Way ANOVA with group and time as fixed factors. The interaction term between dose and timepoint was included in the model. The animals were repeated over the time, and a Tukey adjustment was performed. All statistical tests were two-sided, the nominal level of statistical significance was set to α = 0.05 for effect size estimates, α = 0.01 for normality tests, and α = 0.10 for interaction terms. Analyses were conducted using SAS v9.4^®^ (SAS Institute Inc., Cary, NC, USA).

## 3. Results

### 3.1. Selection of the Base Sequence and Design of the Pre-F Constructs

#### 3.1.1. Selection of the Base Sequence for the Pre-F Designs

The hMPV virus has four subtypes: A1, A2, B1, and B2. To maximize the effectiveness of our vaccine, we first determined the prevalence of the circulating subtypes by analyzing the number of strains of each subtype with known isolation times deposited into the NCBI database from 1982 to 2020 (Figure S1). The total numbers of sequences with isolation dates before 2003 were very low (10 per year or less), representing the retrospective testing of archived samples following the 2001 identification of hMPV. In recent years, detection of subtype A2 predominated, followed by B2 and B1 at lower prevalence levels, while subtype A1 has not been detected. Although the percent diversity between the hMPV F proteins at the amino acid level is only ~6% [34], with few differences within known neutralizing epitopes and high levels of cross-neutralization reported between the subtypes [43], we based our vaccine candidate designs on the most prevalent subtype, selecting the prototypical A2 CAN97-83 strain F sequence (GenBank AY145296).

#### 3.1.2. Design of the Pre-F Constructs

We utilized three strategies to stabilize our designs in the pre-F conformation. The first was hydrophobic cavity filling, which increased the energy barrier to unfolding from the pre-F to the post-F state and enhanced the packing of the hydrophobic core, exemplified by the K138F mutation. The second was intraprotomer interface stabilization, such as the N46V_T160F design, where the two mutations were introduced within a protomer to hold the two positions together by hydrophobic interactions. The residues 46 and 160 interact with each other only in the pre-F conformation, and thus, the mutations N46V_T160F stabilize the pre-F conformation over the post-F conformation. The third was interprotomer interface stabilization, for example the K362F_G366F mutations, which introduced stronger hydrophobic interactions between two neighboring protomers, predominantly in the pre-F conformation. In all the designs, to generate soluble recombinant proteins, the transmembrane and cytoplasmic domains were replaced with the T4 foldon trimerization motif with purification tags added to its C-terminus. Lastly, to enhance serine protease cleavage between the F1 and F2 subunits, the native “RQSR” protease site was replaced with the furin cleavage site “RRRR” [25]. A total of 20 pre-F stabilized designs were generated.

In addition, three benchmarks were produced. The first was the prototypical, subtype A1 based, pre-F A185P construct that was stabilized in the pre-F conformation via the introduction of a proline “helix breaker” residue [25]. As this construct was made in a subtype A1 strain background, to permit most direct comparison with our designs, we introduced the corresponding proline into the same A2 CAN97-83 F used for our designs, generating the D185P construct. Lastly, the subtype A1 based post-F construct was engineered [26].

### 3.2. Production and Biophysical Characterization of the Pre-F Constructs and Benchmark

#### 3.2.1. Yields and Purity

**Figure 1:**
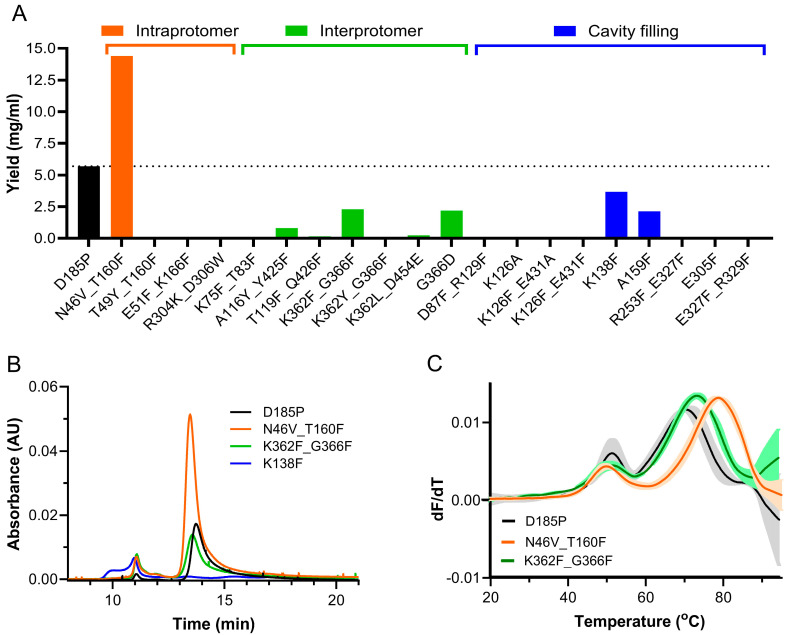
Expression and characterization of human metapneumovirus (hMPV) fusion protein (F) designs. (**A**) Expression levels of the 20, prefusion (pre-F) stabilized, candidate designs and the benchmark. Colors indicate the stabilization strategy used. Dashed line indicates expression level of the D185P benchmark. (**B**) Size-exclusion chromatography (SEC) traces of the pre-F benchmark and the three highest yielding designs (N = 3). (**C**) Thermograms comparing the melting temperatures (Tm) of the benchmark and the two designs with >90% trimer. Tm and colloidal stability were determined by nano differential scanning fluorimetry (N = 3).

Figure 1A summarizes the specific mutations and stabilization strategies of the twenty A2 based designs and compares their yields to the A2 based pre-F benchmark. In all, 6 of the 20 designs (at least 1 for each stabilization strategy) showed some level of expression, with the N46V_T160F construct achieving a yield of 14.4 mg/L, which is considerably higher than the pre-F benchmark, while the K138F and K362F_G366F designs achieved the next highest expression. The stabilization strategies, as exemplified by the highest yielding constructs and the D185P benchmark, are illustrated in Figure S2.

The highest yielding design from each stabilization strategy was next assessed by size-exclusion chromatography (SEC) for trimer percentage and aggregation (Figure 1B), and by SEC-MALS for absolute molecular weight (Table 1). The pre-F benchmark yielded the expected molecular weight, with a well-defined trimer peak amounting to >90% and minimal aggregation. Of our designs, N46V_T160F also showed >90% trimer, while K362F_G366F was only 77% in the trimeric state with notable aggregation, and K138F was entirely aggregated and was not further evaluated.

#### 3.2.2. Thermostability Assessments

The melting temperatures and colloidal stability of the A2 pre-F benchmark D185P and the two designs, K362F_G366F and N46V_T160F, were next determined by nano differential scanning fluorimetry. The major thermal event for the control D185P was recorded at 70.4 °C. The interprotomer design K362F_G366F showed a right shift to a higher melting point of 73.8 °C, while the intraprotomer design N46V_T160F showed an even higher increase in Tm, with the major thermal event recorded at 79.3 °C (Figure 1C).

#### 3.2.3. Antigenicity Assessments

The antigenicity of the K362F_G366F and N46V_T160F constructs, and of the three controls, was then investigated using bio-layer interferometry (BLI) to determine binding to a panel of conformation-specific and linear epitope antibodies. The panel included pre-F specific mAbs to sites Ø (mAb SAN32-2 [40]) and V (mAb SAN27-14 [40]), the strongly pre-F preferred site III (mAb MPE8 [44]), the strongly post-F preferred mAb DS7 to site I [41], and the common region binding mAbs 338 to site II [42] and 101F to site IV [45]. Binding of these mAbs to our N46V_T160F and K362F_G366F constructs was identical to that of the pre-F benchmarks A185P and D185P and differed from the post-F control (Table 2).

### 3.3. Immunogenicity in the Mouse Model

#### 3.3.1. Assessments of Binding, Neutralizing, and Site-Specific Ab Titers

**Figure 2:**
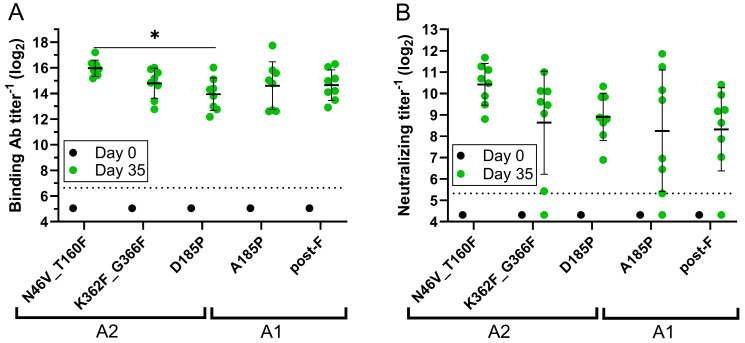
Immunogenicity of pre-F constructs and benchmarks in mice. Groups of eight mice were intramuscularly immunized with 0.5 µg of the indicated hMPV F constructs adjuvanted with Alhydrogel and boosted with the same regimens 21 days post-prime. Blood was collected just before the first immunization (Day 0) and 2 weeks after the second immunization (Day 35). (**A**) hMPV pre-F binding antibody (Ab) titers determined by ELISA. (**B**) Neutralizing Ab titers determined by microneutralization against hMPV subtype A2 strain expressing GFP. Day 0 samples were run as group pools. Dashed lines represent lower limits of quantification. Error bars represent one geometric standard deviation from the geometric mean. * = *p* < 0.05. Brackets below the graphs indicate the subtypes of the base strains of the constructs.

A mouse study was next performed to compare the immunogenicity of the N46V_T160F and G366F_K362F constructs to the D185P benchmark. As this proline mutation was originally described in the A1 background, the A185P (A1 based) pre-F benchmark was also included to ensure full comparability to published data, as was the A1 based post-F benchmark. The hMPV post-F benchmark has been reported to be a mixture of both pre-F and post-F [26] and was therefore heat treated at 70 °C for 10 min to fully convert it to the post-F state [25] prior to inoculation into the mice. Nevertheless, the A1 based pre-F and post-F constructs induced similar levels of binding (Figure 2A) and neutralizing (Figure 2B) Ab titers, possibly due to the dense glycosylation at the apex of hMPV pre-F, which shields site Ø from the immune response [25]. This shielding has been proposed to result in a broader nAb response across all hMPV antigenic sites than is the case with RSV [25], where site Ø is immunodominant. Among the A2 constructs, N46V_T160F induced a 4.1-fold, statistically significantly higher (*p* = 0.0287) geometric mean binding Ab titer and a 2.9-fold higher geometric mean nAb titer on Day 35 than the D185P benchmark, although the latter difference did not achieve statistical significance. The geometric means of the binding and neutralizing Ab titers induced by N46V_T160F were likewise higher than those induced by the A185P benchmark. Further, the N46V_T160F immunized animals showed least variability in both binding and nAb titers, as compared to other groups.

**Figure 3:**
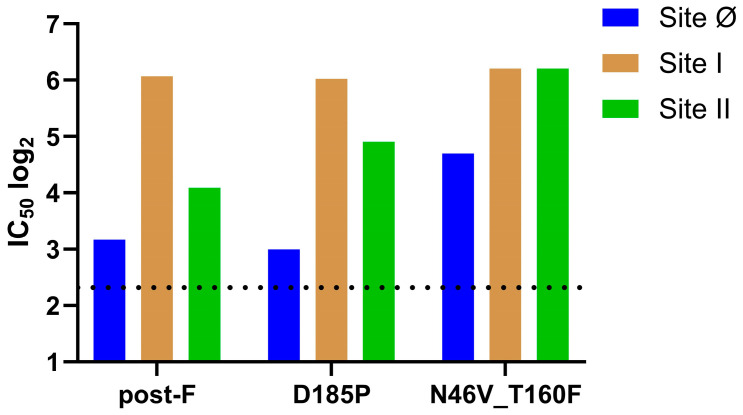
Site specific antibody competition of murine sera. ELISA plates coated with N46V_T160F were incubated with Day 35 serum pools from the indicated groups (eight mice per group). Plates were washed and incubated with biotinylated monoclonal Abs (mAbs), then washed and developed with horseradish peroxidase conjugated anti-Biotin. The 50% inhibitory concentrations were reported as decreases in optical density relative to the control wells receiving only the biotinylated mAbs. The dashed line represents the lower limit of quantification.

To explore the source of the enhanced nAb titers induced by the N46V_T160F construct, the Abs generated in the immunized mice were further characterized in a competitive ELISA. The three anti-hMPV F mAbs selected were: the site Ø binder SAN32-2, the site I binder DS7, and the site II binder 338. The mAbs were competed with Day 35 sera from the N46V_T160F, the D185P, and the post-F immunized groups. Due to volume limitations a single pool was made from the eight mice in each group. All three groups exhibited similar titers of site I competing Abs (Figure 3). However, while D185P immunization induced similarly low levels of site Ø competing Abs as post-F immunization, the titer in the N46V_T160F group pool was ~3-fold higher. Lastly, while the site II titer in the D185P group was slightly (1.8-fold) higher than in the post-F group, immunization with N46V_T160F induced 4.4-fold higher titers than with post-F and 2.5-fold higher than D185P.

#### 3.3.2. Cross-Subtype Neutralization Analysis

**Figure 4:**
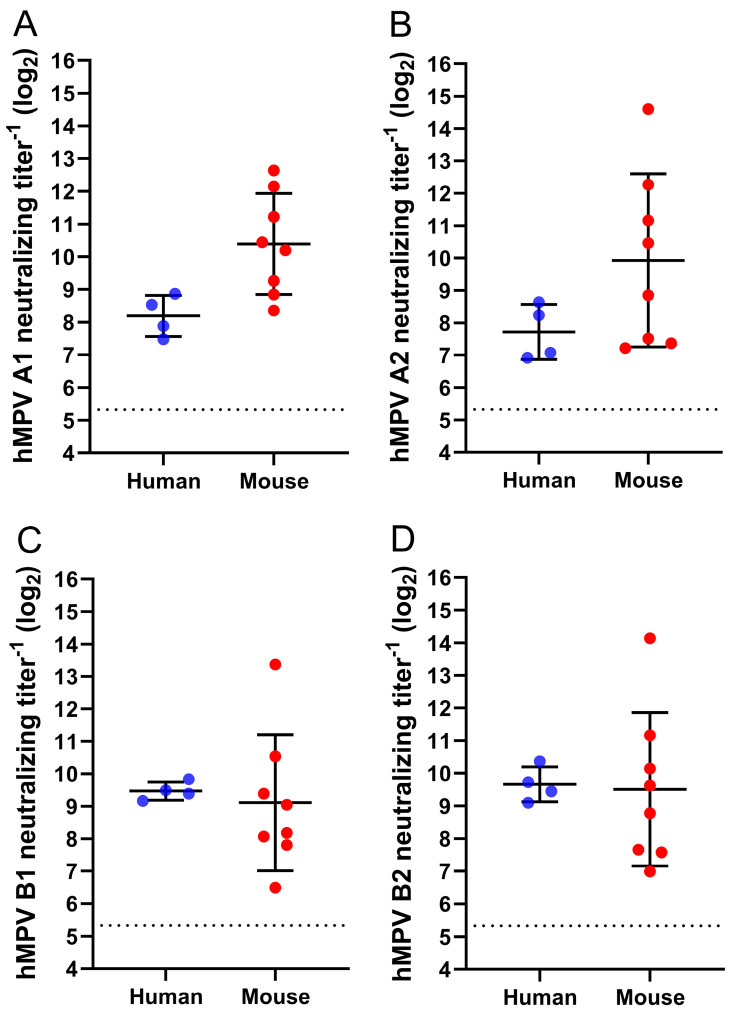
Cross-neutralization assessment of N46V_T160F immunized mouse sera. NAb titers in Day 35 sera of the eight were mice determined against prototype hMPV strains from subtypes (**A**) A1 (NL/1/00), (**B**) A2 (NL/17/00), (**C**) B1 (NL/1/99), and (**D**) B2 (NL/1/94) via PRNT_60_ assays. Four replicates of an in-house generated adult human sera pool were tested in each assay as an intra- and inter-assay control and internal neutralization benchmark. Dashed lines represent lower limits of quantification. Error bars represent one geometric standard deviation from the geometric mean.

As the two genetic lineages of hMPV are, despite ~94% minimum identity in the F protein amino acid sequence, antigenically distinct [32], we interrogated the ability of Day 35 sera from N46V_T160F immunized mice to cross neutralize all four hMPV subtypes. As GFP encoding reporter viruses were not available for all subtypes, all four were interrogated via PRNT_60_ assays utilizing the wild type, prototype virus from each subtype [32]. N46V_T160F immunization induced neutralizing titers in all animals against all four subtypes. In all cases, the geometric mean titer of the mouse sera was similar to, or higher than, that of the adult human serum pool control used as an internal benchmark of virus neutralization sensitivity, as there is no recognized antisera standard for use in hMPV neutralization assays (Figure 4).

### 3.4. Comparison to Next Generation Structure-Based Designs

**Figure 5:**
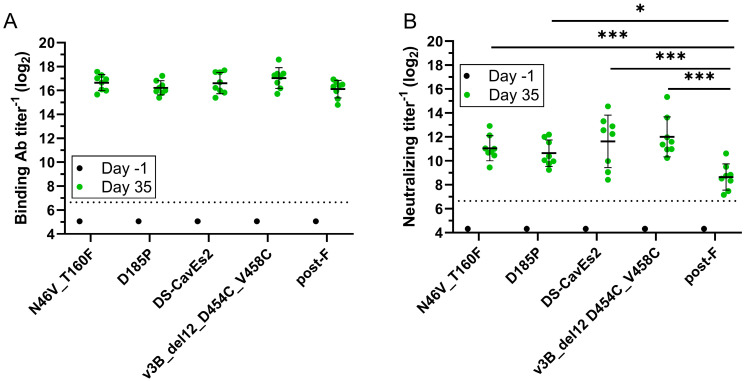
Evaluation of the immune response to the N46V_T160F pre-F construct and additional benchmarks in mice. Groups of eight mice were intramuscularly immunized with 0.5 µg of the indicated hMPV F constructs adjuvanted with Alhydrogel and boosted with the same regimens 21 days post-prime. Blood was collected one day prior to the first (Day −1) and 2 weeks after the second (Day 35) immunization. (**A**) hMPV pre-F binding Ab titers were determined by ELISA. (**B**) NAb titers were determined by microneutralization against hMPV subtype A2 strain expressing GFP. Day −1 samples were run as group pools. Dashed lines represent lower limits of quantification. Error bars represent one geometric standard deviation from the geometric mean. * = *p* < 0.05, *** = *p* < 0.001.

Next, a mouse study was performed to compare the immunogenicity of the N46V_T160F computationally designed construct to the next-generation, structure-based designs v3B_Δ12_D454C-V458C [27] and DS-CavEs2 [11] with the D185P benchmark and the hMPV post-F included as study controls. Of note, DS-CavES2 was produced without furin cleavage to increase trimer percentage (Table S1) but showed similar binding patterns to key mAbs in BLI as a prep produced with furin (Table S2). There were no significant differences in the induction of binding Ab titers by the five constructs (Figure 5A). However, all pre-F constructs induced significantly higher nAb titers than post-F (Figure 5B). The geometric mean nAb titers induced by the pre-F constructs did not differ significantly and those induced by N46V_T160F were within 2-fold of those induced by v3B_Δ12_D454C-V458C and DS-CavEs2.

### 3.5. Long-Term Stability and Epitope Integrity at Higher Temperatures

**Figure 6:**
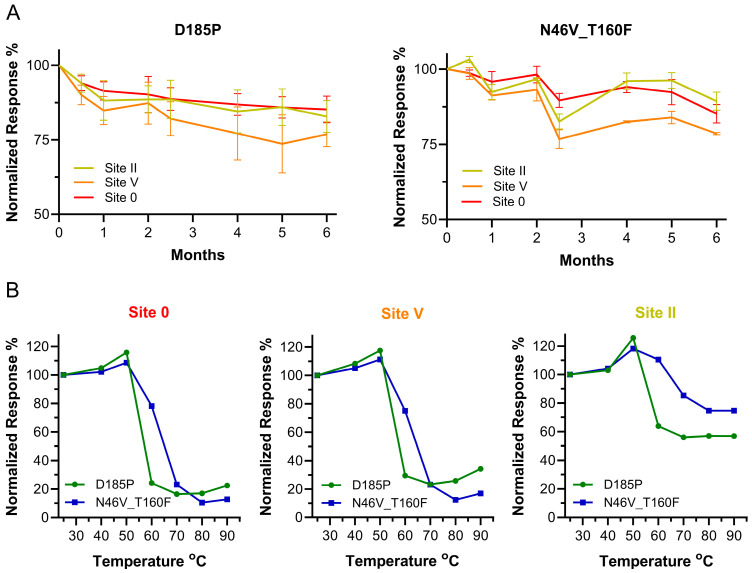
Stability of the pre-F constructs. (**A**) Binding of 338 (site II), San27-14 (site V), and San32-2 (site Ø) IgG to D185P and N46V_T160F stored for up to 6 months at 4 °C measured by bio-later interferometry normalized to 100% at Day 0 (N = 3). (**B**) Binding of San32-2 (site Ø), San27-14 (site V), and 338 (site II) to heat-treated N46V_T160F (blue) and control D185P (green). Samples were heated to the different temperatures shown on the *X*-axis and binding levels were normalized to initial binding levels of untreated samples.

Last, the epitope integrity of hMPV F was examined under two temperature stresses. First, N46V_T160F and D185P, as a control, were stored at 4 °C for up to 6 months. The constructs were aliquoted and the aliquots flash-frozen at intervals, stored at −80 °C, and then all samples were assessed in BLI to evaluate retention of site specific mAb binding (Figure 6A). A panel of three mAbs binding to three different sites was selected, 338 binding to site II, San27-14 to site V, and San32-2 to site Ø, and binding levels were normalized to Day 0 samples. Both constructs retained similar binding of pre-F specific SAN27-14 and SAN32-2 mAbs at 6 months, relative to the Day 0 samples, and showed similar decreases in mAb 338 binding, whose epitope is present on both the pre- and post-F forms. Final retention of binding for D185P was 82.8%, 76.8%, and 85.2% and 89.4%, 78.5%, and 85.2% for N46V_T160F, for sites II, V, and Ø, respectively.

The other temperature stress test on epitope integrity consisted of heating N46V_T160F and D185P to different temperature points and assessing their retention of binding to the same panel of mAbs. Untreated samples were kept at room temperature (25 °C), while treated samples were exposed to temperatures ranging from 40 to 90 °C, increasing by 10 °C. The resulting seven samples for each construct were then exposed to mAbs 338, San27-14, and San32-2, and their binding levels were normalized to the binding of untreated samples (Figure 6B). The largest difference in binding responses between N46V_T160F and D185P was observed at 60 °C for all mAbs, as retention of binding was maintained at 78.2%, 75.0%, and 110.5% for San32-2, San27-14, and 338, respectively (D185P: 24.3%, 29.5%, and 63.9%). At higher temperatures, the two constructs showed a similar binding pattern, except for the slightly improved site II binding for N46V_T160F with 338, a linear epitope antibody.

## 4. Discussion

HMPV was initially isolated and identified as a pathogen in young children in 2001 [7] and first reports of its potential impact on the elderly came out just one year later [1]. Similar to RSV, childhood infection does not protect from re-infection with hMPV, which can cause severe or fatal respiratory disease in older adults and the immunocompromised [46,47,48]. Studies exploring disease burden in the elderly found rates of infection and hospitalization similar to those for RSV and influenza [2,49]. Despite this considerable public health burden, there are currently no licensed vaccines or therapeutics against hMPV, although several are in early stages of development [13,50]. This development is focused on the F protein because of its key function in viral entry and being the primary target of hMPV nAbs, and is further driven by reports that, similar to RSV, stabilization of hMPV F in its pre-F state produces a superior antigen [11,13,27].

Towards addressing this significant unmet healthcare need, we utilized three computational design strategies to generate 20 hMPV vaccine candidates stabilized in the pre-F conformation and extensively characterized them in vitro and in vivo. We found that construct N46V_T160F, generated via intraprotomer interface stabilization, exhibited high yields and trimer percentage, and was the most thermostable. This construct retained all pre-F specific antigenic sites, and the key epitopes remained stable at 4 °C for up to 6 months and showed increased epitope integrity at higher temperatures. Notably, in the mouse model, immunization with N46V_T160F induced 3 to 4-fold higher nAb titers than with either post-F or prototype pre-F benchmarks. Further investigation revealed that this more potent neutralization stemmed in part from increased levels of site Ø and site II competing Abs induced by N46V_T160F immunization, while the less potent site I Ab competing titers were comparable to those induced by the benchmarks. Lastly, sera from N46V_T160F immunized mice neutralized prototype viruses from all four hMPV subtypes.

Interestingly, pre-F and post-F benchmark immunizations induced similar titers of site Ø competing Abs in our study. Site Ø was initially identified on the RSV pre-F [18], where it is the most potent neutralization epitope [21]. Based on structural similarity, the RSV neutralizing sites were assigned to the corresponding sites on hMPV; however, their potency did not necessarily correlate. In particular, the hMPV site Ø is less immunodominant than that of RSV, likely due to its heavy glycosylation [25]. This glycan shield is thought to in part explain the smaller distinction between pre-F and post-F induced nAb titers for hMPV and broader nAb response across all sites than is the case with RSV [40]. Our lead design appeared to enhance exposure of the site Ø and II epitopes, leading to a better immune response.

Recently the McLellan laboratory utilized structure-based design to create a second-generation hMPV pre-F construct (termed DS-CavEs2) which exhibited enhanced expression and melting temperature as compared to their first-generation, proline-stabilized construct we initially utilized as our benchmark. However, these in vitro enhancements did not result in a significant increase in immunogenicity [11]. Structure-based design was likewise utilized by the Kwong laboratory to generate a single-chain, triple-disulfide-stabilized pre-F construct that induced nAb titers >10-fold higher than post-F in naïve mice [27]. When we compared the immunogenicity of our computationally designed N46V_T160F candidate to these next generation structural designs, we observed induction of similar levels of binding and nAbs by all three constructs.

Toward further optimization of N46V_T160F, we are performing cryogenic electron microscopy studies to directly observe the impact of these residues on the trimer structure and test our hypothesis as to how they elicit increased titers of site II competing Abs and how they affect the glycan shield to better expose site Ø. We are utilizing structural biology approaches to build upon this computational modeling design and employing glycan engineering to mask “junk epitopes” from the immune response, following a strategy we utilized to focus the immune response on the most potent neutralizing epitopes of RSV [51].

In summary, N46V_T160F is a promising hMPV vaccine candidate that paves the way for further development and optimization.

## 5. Patents

M.K., A.S., S.S., Y.C., J.A.-S., and L.Z. are inventors on patent WO 2023/102373 A1 that describes the hMPV pre-F antigens reported in this manuscript.

## Figures and Tables

**Table 1:** Trimer percentage and absolute molecular weight of the benchmarks and select pre-F designs.

Sample	Trimer (%) ^1^	MW (kDa) ^2^
D185P	97.1	224.3
N46V_T160F	94.7	266.5
K362F_G366F	76.9	222.8
K138F	0	5860.0

^1^ Trimer percentages as determined by SEC. ^2^ Absolute molecular weights of the major peaks as determined by SEC in line with multi-angle light scattering (SEC-MALS).

**Table 2:** Pre-F stabilized designs exhibit a different pattern of mAb binding than post-F.

mAb	Site	A1 A185P	A1 Post-F	A2 D185P	A2 N46V_T160F	A2 K362F_G366F
San32-2	Ø	**+**	**−**	**+**	**+**	**+**
San27-14	V	**+**	**−**	**+**	**+**	**+**
338	II	**+**	**+**	**+**	**+**	**+**
MPE8	III	**+**	**−**	**+**	**+**	**+**
101F	IV	**+**	**+**	**+**	**+**	**+**
DS7	I	**−**	**+**	**−**	**−**	**−**

Plus sign (+) represents the successful interaction of Immunoglobulin G (IgG) and the protein; a negative sign (−) denotes the lack of binding. The underlined mAbs in the table are pre-F specific or strongly pre-F preferred (N = 3). A1 and A2 before the construct names indicate the subtype of the base strain of the construct.

## Data Availability

The data presented in this study are available on request from the corresponding author as per company policy.

## Supplementary Materials

The following supporting information can be downloaded at: https://www.mdpi.com/article/10.3390/vaccines13050523/s1, Figure S1: Temporal occurrence of the four hMPV genotypes. Figure S2: Illustration of the strategies used to stabilize hMPV F in the pre-fusion state. Table S1: Yield and trimer percentage for DS-CavEs2 produced with and without furin co-transfection. Table S2: mAb binding to batches of DS-CavEs2 produced with and without furin co-transfection.
