# Supplementary material for: A Computationally Designed Prefusion Stabilized Human Metapneumovirus Fusion Protein Vaccine Antigen Elicited a Potent Neutralization Response
Source: Vaccines (Basel). 2025 May 15;13(5):523. doi: 10.3390/vaccines13050523 (PMC12115362; doi:10.3390/vaccines13050523)
Supplement: Supplementary file 1 [file vaccines-13-00523-s001.zip › vaccines-3606535-supplementary.pdf]

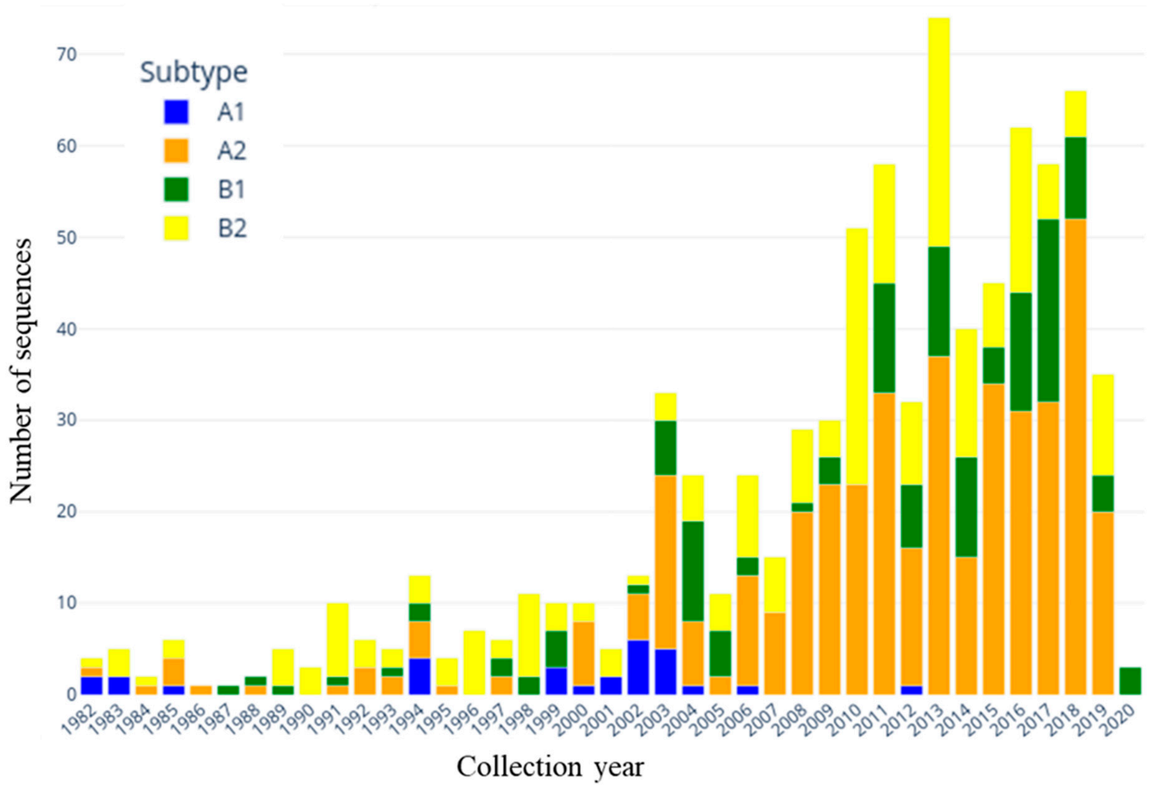

Figure S1. Temporal occurrence of the four hMPV genotypes. Y-axis shows number of strains of each subtype isolated from samples with known collection years deposited into the NCBI database. X-axis shows sample collection years.

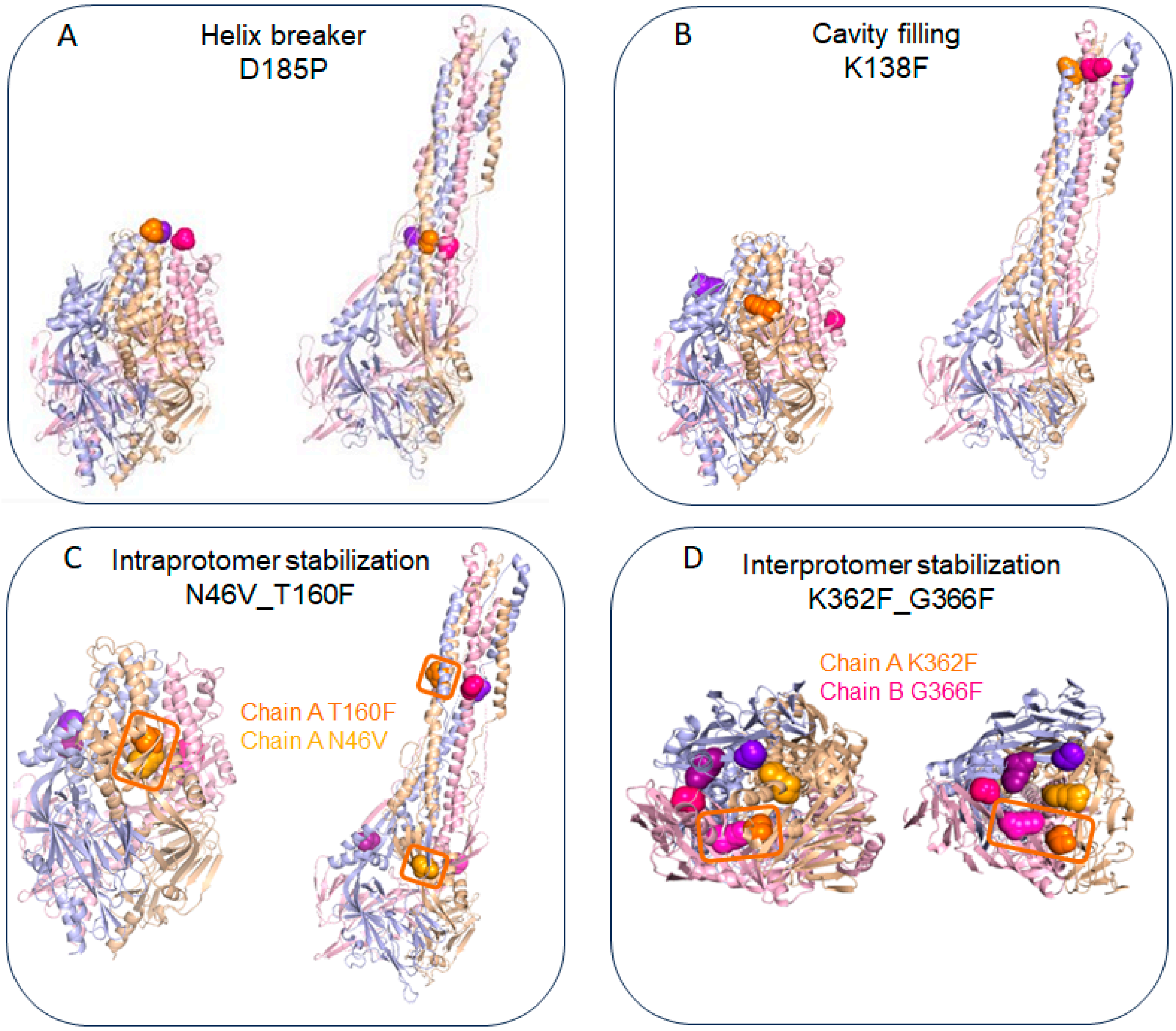

Figure S2. Illustration of the strategies used to stabilize hMPV F in the pre-fusion state. The pre-F state is shown on the left and the post-F state on the right in every panel, with colored globes indicating locations of the residue(s) to be mutated. Side views are shown in panels A – C and a top-down view in panel D. (**A**) Proline helix breaker mutation exemplified by D185P. (**B**) Hydrophobic cavity filling exemplified by the K138F. (**C**) Intraprotomer interfacial stabilization exemplified by N46V_T160F. (**D**) Interprotomer interfacial stabilization exemplified by K362F_G366F.

Table S1. Yield and trimer percentage for DS-CavEs2 produced with and without furin co-transfection.

Parameter	DS-CavEs2 w/o furin	DS-CavEs2 with furin
**Yield (mg/L)**	3.04	1.89
**Trimer %**	96.8	77.9

Shown are yield and trimer percentage of the polished main peak as recorded by size exclusion chromatography.

Table S2. mAb binding to batches of DS-CavEs2 produced with and without furin co-transfection.

mAb	Specificity	A185P	A1 post-F	DS-CavEs2 w/o furin	DS-CavEs2 with furin
**SAN27-14**	**V**	0.69	0.20	0.71	0.76
**SAN32-2**	**0**	0.80	0.28	0.78	0.87
**SAN27-13**	**A** [1]	0.77	0.24	0.74	0.83
**SAN27-40**	**D** [2]	0.56	0.03	0.00	0.00
**SAN27-4**	**post-F**	0.00	1.45	0.00	0.00

Binding levels in nm of a panel of mAbs to the A185P and post-F benchmarks, and to DS-CavEs2 produced with and without furin co-transfection. Specificity refers to the mAb binding sites.

[1] Site A in the interprotomer interface of the trimer [1].
[2] Site D is near the membrane proximal region [1].
Antigens were kept at 5 µg/mL concentration and Abs binding to the biosensors was optimized to 0.6 nm.

References:

1. Rush, S.A.; Brar, G.; Hsieh, C.-L.; Chautard, E.; Rainho-Tomko, J.N.; Slade, C.D.; Bricault, C.A.; Kume, A.; Kearns, J.; Groppo, R.; et al. Characterization of Prefusion-F-Specific Antibodies Elicited by Natural Infection with Human Metapneumovirus. *Cell Rep* **2022**, *40*, 111399, doi:10.1016/j.celrep.2022.111399.
